# Development of Low-Density Polyethylene Films Coated with Phenolic Substances for Prolonged Bioactivity

**DOI:** 10.3390/polym15234580

**Published:** 2023-11-30

**Authors:** Iro Giotopoulou, Renia Fotiadou, Haralambos Stamatis, Nektaria-Marianthi Barkoula

**Affiliations:** 1 Department of Materials Science and Engineering, University of Ioannina, 45110 Ioannina, Greece; i.giotopoulou@uoi.gr; 2 Department of Biological Applications and Technology, University of Ioannina, 45110 Ioannina, Greece; p.fotiadou@uoi.gr (R.F.); hstamati@uoi.gr (H.S.)

**Keywords:** LDPE, carvacrol, thymol, olive-leaf extract, antimicrobial, antioxidant, active packaging

## Abstract

The current study proposes an efficient coating methodology for the development of low-density polyethylene (LDPE) films with prolonged bioactivity for food packaging applications. Three natural phenolic-based substances were incorporated at optimized concentrations in methyl-cellulose-based solutions and used as coatings on LDPE films. The amount of surfactant/emulsifier was optimized to control the entrapment of the bioactive substances, minimizing the loss of the substances during processing, and offering prolonged bioactivity. As a result, the growth of *Escherichia coli* was substantially inhibited after interaction with the coated films, while coated films presented excellent antioxidant activities and maintained their mechanical performance after coating. Considerable bioactivity was observed after up to 7 days of storage in sealed bags in the case of carvacrol- and thymol-coated films. Interestingly, films coated with olive-leaf extract maintained a high level of antimicrobial and antioxidant properties, at least for 40 days of storage.

## 1. Introduction

The integration of bioactive substances into packaging materials has been proposed as a promising strategy for the development of active packaging [1]. The bioactive packaging interacts with the food or its environment to kill the pathogens or reduce the spoilage microorganism growth and slow down the oxidation process. Plant secondary metabolites, such as extracts and essential oils, have a wide spectrum of antimicrobial as well as antioxidant activity and can be potentially used as natural food preservatives [2]. Thymol and carvacrol are volatile phenolic terpenes and major components of essential oils, with documented antimicrobial and antioxidant activity [3]. Similarly, plant extracts, such as mango seed, feijoa skin, wasabi, grape pomace, or skin extracts have been studied for their potential use in active packaging [4,5,6,7] as multicomponent mixtures of bioactive substances [8]. Although olive-leaf extract is known to possess significant anti-inflammatory, antimicrobial, and antioxidant properties due to its high phenolic content, which is comparable to that of olive oil and fruits, its application in active packaging has not been yet optimized [9]. Since olive leaves are a by-product of the olive oil industry, their exploitation as bioactive agents in active packaging applications seems an effective and financially viable solution.

Among the various alternatives for incorporating bioactive substances in packaging materials [10,11] the application of coatings is considered a simple and cost-effective technique. Extrusion and blending, although industrially scalable, can present serious limitations because bioactive compounds are frequently volatile and sensitive to the application of heat [11]. As previously documented [12,13,14,15,16], thermomechanical processing results in considerable loss of phenolic-based compounds and extracts, which varies between 20% and 44% in the case of thymol and carvacrol (Table S1). Thus, contrary to the incorporation via melt-mixing/blending with the packaging material, which is a heat-intensive methodology, coatings offer reduced loss of bioactivity during processing [11]. Other parameters that are essential for the preparation of bioactive films are related to the scalability of the proposed methodology, uniform distribution of the active compound, effect of incorporation on the physical and mechanical performance, direct contact of the active compound with the food product, as well as the release rate of the bioactivity [11,15,17,18,19,20,21,22]. A direct comparison of the coating methodology over thermomechanical processing (extrusion, heat-press, etc.) on critical properties of bioactive films can be found in Table S2. Quite recently, several studies have investigated the incorporation of volatile and non-volatile bioactive compounds in polymer films using the supercritical solvent impregnation technique [23,24,25,26]. The impregnation is normally performed under mild heating conditions (temperatures up to 55 °C); however, the use of supercritical fluids requires additional processing steps, and the bioactivity and mechanical properties of the obtained films are highly dependent on the processing conditions [27].

Based on the above, the coating methodology is still highly relevant for the scale-up of bioactive packaging solutions. Although several studies focus on essential oils or extracts coated on polymeric films, their content varies between 1% and 25% (*w*/*w*) with respect to the weight of the polymer substrate [5,28,29,30,31,32,33,34]. This implies the lack of a standard methodology for the development of coated films with optimum bioactivity. Furthermore, the direct deposition of essential-oil- or plant-extract-based mixtures on the surface of the polymer substrates [6,34,35] has been linked with the poor interaction of the bioactive substance and the substrate, even after the application of surface treatment, and thus with the direct release of bioactive compounds into the food and consequently limited bioactivity over time [36]. The dissolution of the bioactive substances in natural polymer dispersions, such as PLA [29], cellulose derivatives [29], zein [30], alginate, and chitosan [37,38,39] or in synthetic polymers, such as ethylene vinyl alcohol [32] or carboxylated styrene-butadiene latex [7], has been also intensively investigated. This process leads to the formation of an additional layer on the polymer substrate, with doubtful adhesion and possible alteration of the mechanical properties of the substrate. For the large-scale production of the bioactive coated films, the drying stage of the coating should be optimized to avoid the loss of heat-sensitive bioactive compounds. At the same time for the commercialization of bioactive films with phenolic-based substances, it is imperative to develop coatings that function safely and effectively during operation and are stable under different atmospheric conditions [40]. The composition of the proposed coatings should follow the general principles of food regulations. Thus, substances classified as non-toxic, non-hazardous, and not allergen need to be used. It has also been highlighted that it is critical to consider the influence of the active coating on the overall performance of the developed films and ensure that the coating layer is not delaminated either during material conversion steps or during its end use [15]. Till now few works have focused on the functional properties of the films after the application of heating during the drying process [29,30,41,42]. Furthermore, there is not a lot of information on the functional properties of the bioactive films over time under different storage conditions. Most studies on this matter indicate the loss of bioactivity within a few days [36,43].

Based on the above, to prepare effective coatings, solubilization of the bioactive substances must be achieved. Since phenolic-based substances are highly soluble in alcohols, their dissolution was performed in an alcoholic medium (ethanol or methanol). To increase the affinity of the less polar thymol and carvacrol with water, the current research group identified the appropriate type of solvent and the need of using an emulsifier for the preparation of bioactive coatings with wetting ability on LDPE and an antimicrobial response [44]. However, the bioactive solutions using an emulsifier as a reagent resulted in “semi-dry” coatings, which limits their industrial potential. To avoid the evaporation of the highly volatile parts of the coating solutions, methyl cellulose was used for the entrapment of bioactive substances. Methyl cellulose was chosen because of its non-toxic nature along with its thixotropic behavior. Thus, the current study prepared different coating mixtures using methyl cellulose as an edible water-soluble cellulose derivative for the formation of a continuous phase that will host the bioactive compounds and enable their controlled availability. Focus was given to the development and optimization of a sustainable methodology for the incorporation of phenolic-based substances (i.e., carvacrol, thymol, or water-based olive-leaf extract) on LDPE substrate with the goal of creating coated films with prolonged antimicrobial and antioxidant properties for food packaging applications.

The main outcome of this research is that using mild solvents/reagents and optimizing their quantities enabled the evaporation of the solvent, without the extensive loss of the volatile bioactive substances, and supported the drying of the coating under room temperature/moderate heating conditions. The bioactive substance content along with the respective emulsifier concentration were optimized with respect to the antimicrobial properties of the coated films. The bioavailability of the coated films was measured as a function of the storage conditions. Finally, the effect of the functional coatings on the mechanical properties of the prepared films was evaluated and discussed. To the best of our knowledge, this study is the first to report the design of phenolic bioactive coatings on LDPE substrates that result in films with prolonged antimicrobial and antioxidant response (at least 40 days in the case of olive-leaf extract).

## 2. Materials and Methods

### 2.1. Materials

LDPE film (DOWTM LDPE 352E, thickness 70 μm, density 0.925 g/cm^3^) was kindly provided by Achaika Plastics S.A. (Aigio, Greece). Carvacrol (purity ≥ 98%), thymol (purity ≥ 99%), and methyl cellulose (MC) were purchased from Sigma Aldrich, Steinheim, Germany. Tween 80 (Polysorbate 80) was purchased from Sigma Aldrich, Oakville, ON, Canada. Methanol (ACS reagent Purity ≥ 99.8%) and ethanol (Purity ≥ 99.8%) were purchased from Honeywell Fluka, Seelze, Germany. Water-based olive-leaf extract (OLE) has been developed by members of the research team following the methodology presented elsewhere [45].

### 2.2. Methods

#### 2.2.1. Preparation of the Coating Solutions and Coated LDPE Films

**Figure 1:**
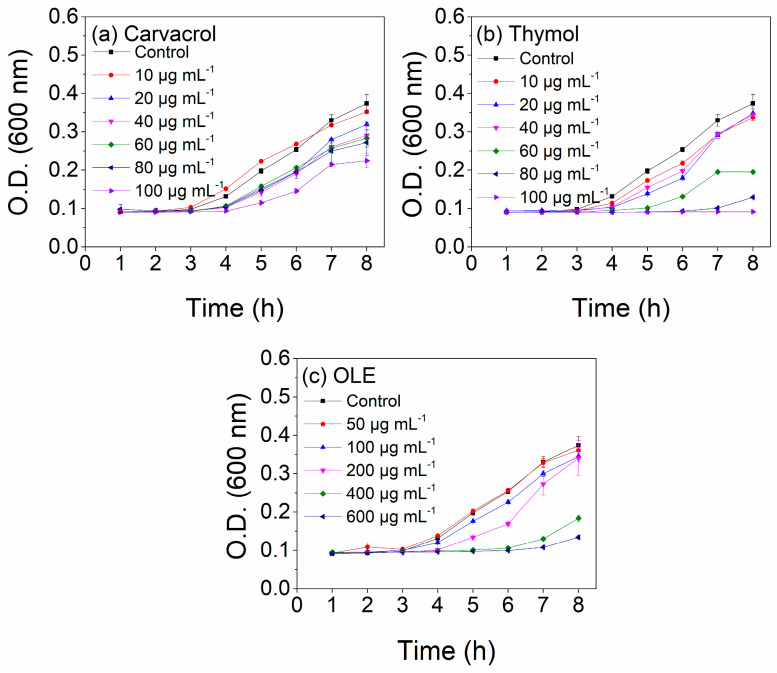
Antimicrobial activity of (**a**) carvacrol-, (**b**) thymol-, and (**c**) OLE-based solutions against gram-negative bacteria *E. coli*.

For this study, 2% *w*/*v* MC with different amounts of Tween 80 (3, 5, 7% *v*/*v*) were dissolved in water by mixing at 80 °C and 400 rpm for 1 h in a closed vial. In a separate vial, 5% *w*/*v* carvacrol or thymol was dissolved in ethanol. In the case of OLE-based coatings, methanol was used as a solvent and the amount of OLE was increased to 20% *w*/*w* based on the antimicrobial response of the bioactive substances (see Figure 1). The type of solvent used to dilute the bioactive substances was based on initial results [44]. An equal amount of solvent (water or alcohol) was used in MC- and bioactive-based solutions. After reaching room temperature, the two solutions were mixed at 400 rpm for 4 min. The same procedure was followed for the preparation of reference solutions (without the addition of any bioactive substance). Blade coating was used to apply appropriate amounts of bioactive solutions on the LDPE substrate. The bioactive content with respect to LDPE weight was set at 5% *w*/*w* loading for carvacrol or thymol and 20% *w*/*w* for OLE. Most of the films were left to dry at room temperature for 24 h. Drying was also performed at an elevated temperature (45 °C) for 5 min, 15 min, and 30 min, respectively. Coated films were tested immediately after drying (same day) and after storage under different conditions. A batch of coated films was sealed in bags immediately after drying, and a second batch was allowed to interact with air (characterized as “close storage” and “open storage”, hereafter). The storage in both cases was performed at room temperature (23 ± 2 °C), while films were placed in a dark room at 50% RH to avoid any deterioration due to light exposure and/or humidity. The antimicrobial and antioxidant properties of the two batches of the coated films were examined at different intervals of storage (up to 40 days in the case of “close storage” and 8 days under “open storage”) to determine the optimum storage conditions.

#### 2.2.2. Microscopy

To examine the morphology of the coatings, a droplet of each solution was placed between two glass slides and placed on the stage of an optical microscope. Images were taken with the help of a camera (Bresser MicroCamLab, Bresser GmbH, Rhede, Germany).

#### 2.2.3. FTIR-ATR

Jasco FT/IR-4700 spectrometer (Jasco, Tokyo, Japan) was used to collect the Fourier transform infrared spectra of the films, with an external device for attenuated reflectance (ATR). Specifically, 184 scans were performed with a resolution of 2 cm^−1^ in the wavenumber range of 600–4000 cm^−1^. The obtained data were evaluated with Spectra Manager Ver.2 14A software (Jasco, Tokyo, Japan). Before any measurement, a background spectrum from the empty cell was received.

#### 2.2.4. TGA Measurements

TGA was performed using a Netzsch-STA 449C Jupiter instrument (Marburg, Germany). The researchers heated 7–10 mg of the substances and the films at a rate of 20 °C/min, under 40 mL/min nitrogen flow from 20 °C to 610 °C.

#### 2.2.5. Antimicrobial Properties

*Escherichia coli (E. coli)* is a bacterium that causes quite severe foodborne illness and is commonly found in several food products such as meat, milk, vegetables, or fruits [46,47]. Since the proposed films are designed for packaging products that may contain this bacterium, *E. coli* BL21DE3 was selected as a good representative of gram-negative strains to assess the antimicrobial properties of the films. The bacterial culture methodology was selected since it can be easily controlled and reproduced at different time intervals. The antimicrobial properties of the films were assessed following a protocol described in more detail elsewhere [48]. In short, fresh bacterial culture was grown in sterile Lysogeny Broth (LB). A bacterial population from the exponential phase was added to 0.9% *w*/*v* NaCl solution (~10^7^ CFU mL^−1^). Approximately 0.25 cm^2^ of each film was added into Eppendorf tubes containing 100 μL of the above bacterial population and incubated for 16 h in a cold chamber. Then, 100 μL of bacteria in the absence of a sample were used as a control. Then, 25 μL of the bacterial population that interacted with the sample were inoculated into 225 μL of fresh sterile LB medium in a 96-well Elisa microplate. The microplate was then incubated at 37 °C under stirring and *E. coli* growth was determined by measuring the Optical Density, O.D. at 600 nm every hour for 8 hours. The antimicrobial efficiency was assessed through the calculation of the lethal effect of the prepared films, which is defined as the percentage growth inhibition of treated cells compared to control at the exponential growth phase [49]. All measurements were performed in triplicate.

(1) $$Lethal\;effect\left(\%\right)=\frac{{O.D.}_{control}-{O.D.}_{sample}}{{O.D.}_{control}}100$$

where O.D._control_ is the optical density at 600 nm of the control, and O.D._sample_ is the optical density at 600 nm of the different samples at the exponential growth phase (3 h after the initial incubation based on the obtained results).

#### 2.2.6. Antioxidant Properties

The antioxidant activity of the prepared films was evaluated based on the DPPH (2,2-diphenyl-1-picrylhydrazyl) radical scavenging assay as described before [50]. A solution of 0.1 mM DPPH in ethanol was prepared. For this study, 10 mg of the different films (approximately 1 cm^2^) were placed in Eppendorf tubes, in which 300 μL of the DPPH solution and 700 μL of ethanol were added to reach a final volume of 1 mL. The mixtures were stirred vigorously and kept in the dark at room temperature for 30 min. The absorbance was measured spectrophotometrically at 517 nm. Ethanol was used as a blank. All experiments were conducted in triplicate and expressed as mean ± standard deviation. The antioxidant efficiency of the films was determined by the decolorization of the ethanol solution of DPPH. DPPH radical scavenging activity was calculated according to the following Equation (1):

(2) $$DPPH\;radical\;scavenging\;activity\left(\%\right)=\frac{A_{control}-A_{sample}}{A_{control}}100$$

where A_control_ is the absorbance of the control, and A_sample_ is the absorbance of the different film samples.

#### 2.2.7. Mechanical Properties

The mechanical properties of the films were assessed using a universal tensile testing machine, equipped with a 2 kN load cell (Jinan Testing Equipment IE Corporation, Jinan, China). According to ASTM D 638-02a, three to five dog-bone V-type specimens were stamped, with a cross-section area of 0.22 mm^2^ and an active length equal to 9.53 mm. The specimens were tensioned at a deformation speed of 30 mm/min at 25 °C. Force (N) and deformation (mm) were recorded during the test. Based on these data and the dimensions of the specimens (gauge dimensions), Young’s modulus (E), yield and tensile strength (σ_y_, σ_uts_), and % strain at break (ε_b_) were calculated.

#### 2.2.8. Statistical Analysis

All analyses were carried out in triplicate, in which the results were recorded as mean ± standard deviation. Student’s *t*-test, one-way ANOVA analysis, and Tukey’s multiple comparison tests were implemented using IBM SPSS Statistics version 21 (SPSS Inc., Chicago, IL, USA).

## 3. Results and Discussion

### 3.1. Optimization of the Prepared Bioactive Mixtures and Performance of LDPE-Coated Films Immediately after Drying

The first goal of the current study was to identify the appropriate concentration of the different bioactive compounds in selected solvents (ethanol for carvacrol/thymol and methanol for OLE) to achieve sufficient inhibition of *E coli* growth. As observed in Figure 1, thymol offers the highest inhibition against *E. coli* growth followed by carvacrol, while a much higher concentration of OLE is required to achieve high levels of *E. coli* inhibition. The antimicrobial activity of the terpenes and the olive-leaf extract against the gram-negative bacteria depends on the dosage. More specifically, from the onset of the exponential phase, the growth of *E. coli* is inhibited especially for concentrations higher than 80 μg/mL of carvacrol or thymol and 400 μg/mL of OLE. Since OLE is a mixture of phenolic and non-phenolic compounds, its inhibition ability against *E. coli* is lower than the respective inhibition of carvacrol/thymol [44]. Based on the above and with the aim to obtain similar levels of inhibition from the prepared coatings, the final concentration of carvacrol-/thymol-based coating was set at 5% *w*/*w* and 20% *w*/*w* of OLE with respect to the weight of LDPE.

**Figure 2:**
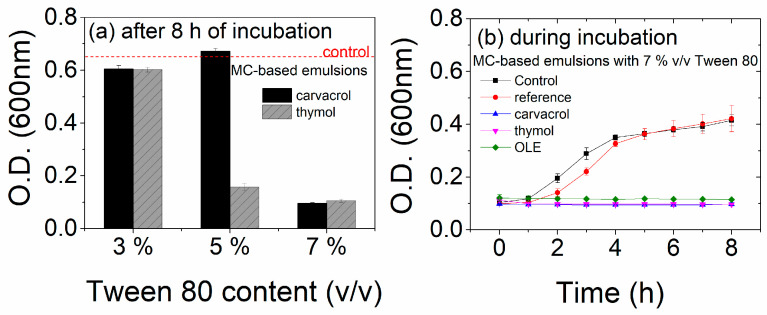
Antimicrobial properties of LDPE films coated with MC-based emulsions: (**a**) effect of Tween 80% *v*/*v* content on the O.D. after 8 h of incubation, (**b**) antimicrobial response of all coated films during incubation at optimal Tween 80 *v*/*v* content (7%). Note that the control refers to the *E. coli* growth without any sample, while the reference sample refers to LDPE-coated films with MC/Tween 80-based emulsions (without the addition of any bioactive substance).

The next goal of the current study was to define the composition of the coating solution to enable its quick drying without substantial loss of bioactivity. As shown in a preliminary study, coatings prepared purely with the bioactive substance and water/alcohol as solvent did not show any inhibition against *E. coli*, and this was mainly linked with the evaporation of the solvent during the drying process and the subsequent loss of the volatile bioactive substances (mainly in carvacrol- and thymol-based solutions) [44]. The use of Tween 80/glycerol was suggested as a potential solution to improve the antimicrobial response of the coatings [32]. This study concluded that the inhibition of *E. coli* was improved. However, this process resulted in “semi-dry” coatings which indicate the need for optimization of the Tween 80 content. Thus, the addition of a reagent that will enable the drying of the coating without the loss of bioactivity is also desirable. In this context, mixtures containing MC as a continuous phase and different Tween 80 concentrations were prepared with carvacrol and thymol and applied as coatings on LDPE films. The antimicrobial response of these films after eight hours of incubation is presented in Figure 2a. Figure 2b presents the antimicrobial performance of LDPE-coated films for all three bioactive substances for mixtures prepared with 7% *v*/*v* Tween 80 content.

As observed in Figure 2a, Tween 80 has a great impact on the antimicrobial properties of the coated films. Almost no inhibition is observed at low Tween 80 concentrations (3% *v*/*v*), while considerable inhibition is observed at 7% *v*/*v* content. An intermediate amount of Tween 80 (5% *v*/*v*) is sufficient to inhibit *E. coli* growth only in the case of thymol-based coatings. Since 7% *v*/*v* is the optimum concentration, the antimicrobial properties of the films during 8 h of incubation with *E. coli* were evaluated as well (Figure 2b). Coated LDPE films with MC and 7% *v*/*v* Tween 80 without any bioactive compounds were also examined for comparison reasons. All films were examined immediately after drying at room temperature. Results indicate that all bioactive-coated films can inhibit the growth of *E. coli* since the optical density at 600 nm is constant and close to zero during the 8 hours of measurement. On the contrary, reference samples follow the behavior of the control sample, which suggests that LDPE, Tween 80, and/or MC do not have any inhibition capability against *E. coli*.

**Figure 3:**
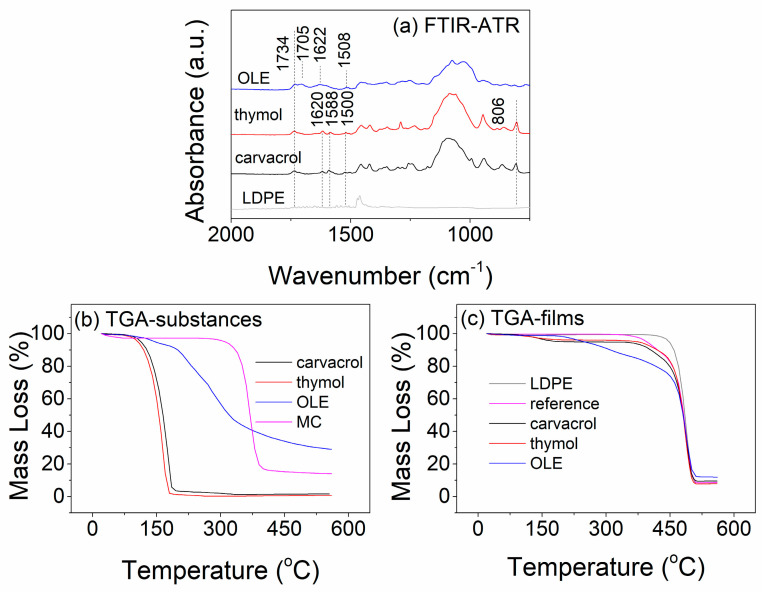
(**a**) FTIR-ATR spectra of LDPE uncoated and coated films with bioactive substances, (**b**) TGA curves of bioactive substances and MC, and (**c**) TGA curves of LDPE uncoated and coated films with bioactive substances. Note that the reference sample refers to LDPE-coated films with MC/Tween 80-based emulsions (without the addition of any bioactive substance).

The presence of the bioactive substances on the surface of the coated LDPE films and their amount/thermal stability after coating were verified by FTIR-ATR, and TGA measurements, respectively. As observed in Figure 3a, all bioactive coated films present a new characteristic peak at 1734 cm^−1^, which can be ascribed to the stretching vibration of the C=O bond of Tween 80 [51]. Regarding LDPE coated with carvacrol/thymol, new peaks appear at 1620 cm^−1^, 1588 cm^−1^, and 1500 cm^−1^, which can be attributed to the stretching vibration of C=C bonds of carvacrol or thymol, while the new peak at 806 cm^−1^ can be assigned to aromatic C-H bending [26,52]. New peaks also appear at 1705 cm^−1^, 1622 cm^−1^, and 1508 cm^−1^ in LDPE-OLE-coated films. According to the literature, the peak at 1705 cm^−1^ can be ascribed to the stretching of the C=O bond of oleuropein and/or other flavonoids that are present in the olive-leaf extract [53]. The peak at 1622 cm^−1^ can be assigned to the characteristic vibration of the carbonyl group [54], and the peak at 1508 cm^−1^ can be assigned to C=C stretching. The above characteristic peaks of the bioactive compounds, which clearly appeared in the spectra of the coated films after drying, confirm the successful incorporation of the coatings on the LDPE substrate.

The thermal stability of the bioactive substances and MC, as well as that of LDPE-uncoated and coated films, is presented in Figure 3b,c, respectively. As observed in Figure 3b, the thermograms of carvacrol and thymol remain stable up to app. 100 °C, while a steep mass loss (app. 100% of the initial weight) is obtained between 100 and 200 °C. OLE also starts to lose weight at app. 100 °C; however, its weight loss curve is more gradual and does not reach a plateau. At the end of the experiment (app. 560 °C), the residual weight of OLE is app. 30%. The main weight loss of MC starts at app. 260 °C and is completed at 425 °C. These results clearly indicate the volatile character of carvacrol, and thymol compared to OLE. The remaining weight in the case of OLE can be attributed to the presence of solid phases and minerals such as K, Ca, S, P, and Mg [55,56]. Based on the results of Figure 3c, it can be stated that the bioactive compounds present higher thermal stability when applied as coatings since their release appears at higher temperatures (15–80 °C) compared to those obtained when tested in their pristine form (Figure 3b). More specifically, the main weight loss starts at app. 115 °C in the case of films coated with carvacrol and thymol, while the respective loss appears at 180 °C in OLE-based coatings. Based on this information, it is possible to calculate the weight content of the bioactive substances after the drying process. Uncoated LDPE films show substantial weight loss at temperatures above 400 °C. Respective loss in reference films (LDPE coated with MC/Tween 80) appears above 335 °C. Thus, weight loss up to 335 °C can be attributed to the release of bioactive substances. The calculated weight content of the bioactive substances is app. 4.5% for carvacrol, 3.7% for thymol, and 12.0% for OLE. The weight content of the volatile substances (carvacrol and thymol) is very close to that incorporated during the preparation of the coating, indicating minimal loss of the volatile substances during the drying process. In the case of OLE, the weight loss of 12.0% can be associated with the release/degradation of the volatile part of the extract. Compared to the reference/LDPE films, the remaining weight (app. 4.5%) is higher in the case of OLE-coated ones. This weight is associated with the remaining OLE in the coated film in the form of solid residues. Thus, the total OLE content is estimated at app. 16.5% *w*/*w*. Furthermore, it is important to note that all bioactive coatings lose less than 0.3% of their weight up to 50 °C, which is normally the temperature used for drying in industrial environments.

These findings can be supported by optical microscopy images of the coating mixtures (Figure S1), which reveal the presence of spherical droplets for both carvacrol- and thymol-coating emulsions, with the heat-sensitive bioactive compounds being entrapped inside the observed micelles. During the heating process of the coatings, the continuous phase of the emulsions evaporates, while the heat-sensitive compounds remain intact under the protection of the micelles. On the other hand, Figure S1 reveals the presence of a non-dissolved solid phase in the form of agglomerates in OLE-based coatings. To confirm that the application of heat during the drying process does not result in excessive release of the bioactive substances and in turn loss of bioactivity, the antimicrobial activity of all coated films was evaluated as a function of the duration of drying at elevated temperatures. As observed in Figure S2, drying of the coatings at 45 °C results in films with high antimicrobial performance since all coated films present limited *E. coli* growth after 8 h of incubation. Comparing these results with the respective ones after room temperature drying (Figure 2b), it can be deduced that there is almost no loss of bioactivity during drying at 45 °C (observed differences lie within the experimental error). These findings are in line with the TGA observations where films demonstrated limited mass loss during heating up to 50 °C. Thus, in conjunction with the FTIR-ATR, it can be concluded that the developed coatings have been successfully applied on the surface of the LDPE substrate and the incorporation of the bioactive compounds in MC/Tween 80, leads to bioactive compounds with high thermal stability.

**Figure 4:**
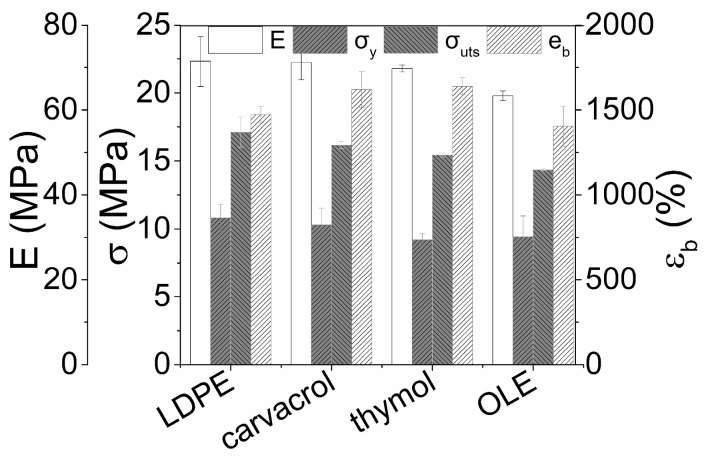
Mechanical properties (E, σ_y_, σ_uts_, ε_b_) before and after the application of carvacrol-, thymol- and OLE-based coatings on the surface of LDPE films.

It is well accepted that LDPE films find use as packaging materials since their mechanical properties are adequate for such applications [57]. It is, therefore, of crucial importance to develop coated films with minimum effect on key mechanical properties (E, σ_y_, σ_UTS_, and ε_b_) of the LDPE substrate. This effect has been assessed and is presented in Figure 4. A limited decrease is observed in E, σ_y_, σ_UTS_, in coatings with carvacrol or thymol, while their strain at break is enhanced by 10% with respect to the uncoated LDPE one. This finding has been reported in the literature when MC/essential oil mixtures have been applied as coatings on LDPE substrates [58]. On the contrary, the films coated with OLE present an 11–16% drop in Young’s modulus and yield/tensile strength and an app. 5% reduction at the elongation at break. The enhancement in the elongation at the break of carvacrol/thymol-coated films could be possibly linked with the minimization of surficial defects in the presence of the MC-based coatings [44]. The reduction of the mechanical properties after incorporating plant extracts in polymer substrates has also been mentioned in the literature [55] and could be associated in the case of OLE-based coating with the presence of solid residues after the drying process. These residues could result in the formation of stress concentration points and the respective reduction of all mechanical properties. Even so, the observed deterioration in OLE-based coatings is quite limited and does not entail any risk to the applicability of the coated films at a commercial level.

### 3.2. Bioactivity of the Coated Films under Different Storage Conditions

All coated films with bioactive compounds present almost total inhibition of *E. coli* immediately after drying, thus, the next crucial question is on the capability to maintain their bioactivity for a prolonged duration under different storage conditions. Since oxidation and microbial growth are the main factors that deteriorate food quality, the present work focuses both on the antimicrobial and the antioxidant properties of the coated films.

**Figure 5:**
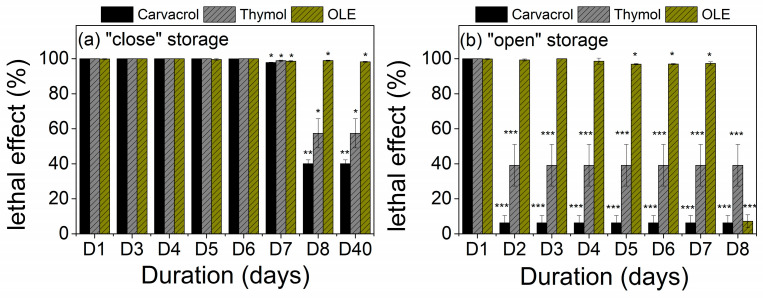
Antimicrobial properties of bioactive-coated films under different conditions and duration (days) of storage; (**a**) “close” storage, (**b**) “open” storage against gram-negative bacteria *E. coli*. The lethal effect is defined as the percentage growth inhibition of *E. coli* cells compared to a control sample at the exponential growth phase (5 h) (* *p* < 0.05, ** *p* < 0.01, *** *p* < 0.001).

Results of the antimicrobial properties as a function of storage conditions are illustrated in Figure 5. As observed in Figure 5a, the bactericidal effect of films with thymol- and carvacrol-based coatings remains constant for up to 6 days and diminishes after 8 days of “close” storage. Interestingly, OLE-based films present considerable inhibition of *E. coli* growth even after 40 days of storage in sealed bags. On the contrary, when films are left to interact with the environment, their inhibition capability against *E. coli* diminishes within 3 days in the case of LDPE with thymol- and carvacrol-based coatings, while OLE-based films preserve the bactericidal effect up to 8 days of exposure (Figure 5b). These findings can be associated with the abrupt release of the volatile part of the bioactive substances when exposed to an open environment. Overall, it can be assumed that in the sealed environment, the gradient in the concentration of the bioactive substances between the coating and the air is small, and this results in reduced flux and in turn lower diffusion of the bioactive substance and prolonged release of the bioactivity. The higher stability of the OLE-based coatings is associated with the non-volatile solid residues of the extract that are not diffused in the air and are stable over time displaying statistically significant differences in comparison to the thymol and carvacrol-based coatings (Figure S3).

**Figure 6:**
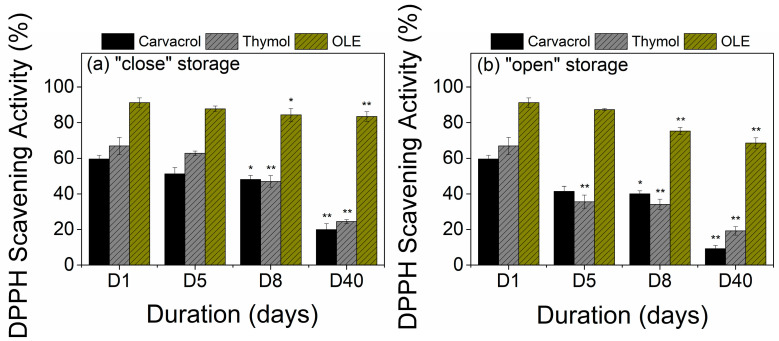
DPPH Scavenging activity of bioactive-coated films under different conditions and duration (days) of storage; (**a**) “close” storage, (**b**) “open” storage (* *p* < 0.05, ** *p* < 0.01).

Results of the antioxidant properties of the coated films as a function of storage conditions are illustrated in Figure 6. Uncoated LDPE films and LDPE-coated films with MC/Tween 80-based emulsions without the addition of any bioactive substance do not show any antioxidant activity, and for this reason, they are not included in the illustrations. As observed in Figure 6, all films present high antioxidant activity (>60%) just after manufacturing. Thymol-based coatings present similar antioxidant responses to carvacrol-based ones; however, OLE-based coatings present extremely high DPPH scavenging activity (app. 90%). This finding is linked to the already documented antioxidant efficiency of the phenolic components in olive leaves [9,59]. In line with the antimicrobial response, the reduction in the DPPH scavenging activity after “close” storage is lower compared to the “open” one, with up to a 25% drop in carvacrol/thymol-based coatings, and 7% in OLE-based ones on day 8. Films with OLE managed to maintain a high level of DPPH scavenging activity (almost 85%) after 40 days of “close” storage presenting statistically significant differences between the coated films (Figure S4). The maintenance of higher antioxidant activity after “close” storage can be attributed to the lower release of the volatile part of the bioactive substances under “sealed” conditions, and in the case of OLE with the high non-volatile content of the bioactive compound. The antioxidant activity of films stored in open space is reduced as the duration of storage increases, and this is more pronounced in films with carvacrol/thymol-based coatings, which present app. 40% drop compared to OLE-based ones that show up to 20% drop within the first 8 days of storage. Interestingly, further exposure (40 days) leads to an 80% reduction of the scavenging activity in carvacrol/thymol films and only a 25% drop in OLE-based films compared to day 1. Overall, it seems that the antioxidant activity is better preserved in OLE-based films, making these films ideal for food applications where lipid oxidation prevails.

Based on the obtained results, it can be concluded that the prepared coatings, especially those containing OLE, are superior to other antimicrobial coatings applied on an LDPE substrate [11,60,61,62] since they present considerably longer maintenance of their antimicrobial response under sealed conditions. The scavenging activity of OLE-based coated films is also superior to similar materials reported in the literature [16,33,42,57,59]. Thus, the main advantage of the current study is that the composition of the coating layers has been optimized to offer prolonged bioactivity at lower bioactive contents compared to previous studies, both in terms of antimicrobial [28,63] as well as antioxidant response [33,42,57,59], following industrially relevant processes. Based on their high antimicrobial response, OLE-based coated films could be used as packaging materials, in food categories, such as meat, vegetables, or fruits, which suffer from *E. coli* growth. Similarly, the high oxygen scavenging capacity of these films supports their potential for the packaging of products that are sensitive to oxidation. Thus, meat products, and especially those with high polyunsaturated fatty acid content, are good candidates to explore both the antimicrobial and antioxidant potential of OLE-based coated LDPE films. Further work on fungal strains and actual food products should be performed to fully assess the perspective of the proposed films under a variety of microorganisms and in vivo.

## 4. Conclusions

The current study focuses on the incorporation of phenolic-based substances on LDPE substrate aiming to create coated films with prolonged bioactivity for food packaging applications. It can be concluded that through the control of the amount of emulsifier/MC, it was possible to create bioactive emulsions, that entrapped the volatile and water-insoluble substances, which resulted in their protection during the drying process. Coatings based on heat-stable substances such as OLE, presented higher stability over evaporation during the drying process due to their high content of solid phase. Apart from their high thermal stability, the developed films presented high antimicrobial and antioxidant efficiency, without significant alteration of their mechanical properties. Although the bioactive substances diffuse over time (during storage), it was confirmed that both antimicrobial and antioxidant properties were maintained for prolonged durations under sealed storage conditions. The prepared coatings are superior to other bioactive coatings applied on LDPE substrate since they present considerably longer maintenance of their bioactivity, which is more pronounced in the case of the antioxidant response of OLE-based coatings (85% DPPH scavenging ability after 40 days of storage). Based on the above, the key innovation of the current study is that it proposes, for the first time, a sustainable methodology for the development of phenolic-based coatings with prolonged bioactivity. This methodology could be easily industrialized and transferred to other polymer substrates for the development of active flexible packaging.

## Data Availability

Data are contained within the article and Supplementary Materials.

## Supplementary Materials

The following supporting information can be downloaded at https://www.mdpi.com/article/10.3390/polym15234580/s1: Table S1: Effect of thermomechanical processing on the thermal stability of bioactive compounds; Table S2: Main differences of the coating technology and the thermomechanical processing; Figure S1: Optical microscope images of (a) carvacrol-, (b) thymol- and (c) OLE-based mixtures (with MC/Tween 80); Figure S2: Antimicrobial properties of LDPE bioactive-coated films with MC-based emulsions after drying at 45 °C for 5 min, 15 min and 30 min. Red line refers to the control, *E. coli* growth without any sample; Figure S3: Comparison of the antimicrobial response of carvacrol-, thymol- and OLE-based films at critical time intervals for “close” and “open” storage conditions; (a) “close” storage—day 8, (b) “open” storage—day 2, against gram-negative bacteria *E. coli*. The lethal effect is defined as the percentage growth inhibition of *E. coli* cells compared to a control sample at the exponential growth phase (5 h) (* *p* < 0.05, ** *p* < 0.01, *** *p* < 0.001, **** *p* < 0.0001); Figure S4: Comparison of the DPPH Scavenging activity of bioactive-coated films at critical time intervals for “close” and “open” storage conditions; (a) “close” storage—day 40, (b) “open” storage—day 40. (* *p* < 0.05, ** *p* < 0.01, *** *p* < 0.001, **** *p* < 0.0001).
