# Supplementary material for: Development of Low-Density Polyethylene Films Coated with Phenolic Substances for Prolonged Bioactivity
Source: Polymers (Basel). 2023 Nov 30;15(23):4580. doi: 10.3390/polym15234580 (PMC10707956; doi:10.3390/polym15234580)
Supplement: Supplementary file 1 [file polymers-15-04580-s001.zip › polymers-2706991-supplementary.pdf]

polymers

MDPI

Article

Supplementary Material

On the development of low-density polyethylene films coated with phenolic substances for prolonged bioactivity

Iro Giotopoulou [1], Renia Fotiadou [2], Haralambos Stamatis [2] and Nektaria - Marianthi Barkoula [1,*]

[1] Department of Materials Science and Engineering, University of Ioannina, GR-45110, Ioannina, Greece; i.giotopoulou@uoi.gr (I.G.), nbarkoul@uoi.gr (N.-M.B.)
[2] Department of Biological Applications and Technology, University of Ioannina, GR-45110, Ioannina, Greece; p.fotiadou@uoi.gr (R.F.), hstamati@uoi.gr (H.S.)
* Correspondence: nbarkoul@uoi.gr; (N.-M.B.); Tel.: (+30 26510 08003)

Table S1. Effect of thermomechanical processing on the thermal stability of bioactive compounds

Bioactive substance	Processing method	Temperature [°C]	Mass loss of bioactive compound (%)	Reference
Thymol and Carvacrol	Hot press	190	25-44	[12]
Linalool or methylchavicol	Blown film extrusion	160	97	[13]
Carvacrol	Blown film extrusion	150	34	[14]
Thymol	Blown film extrusion	150 °C	20	[14]

Table S2: Main differences of the coating technology and the thermomechanical processing

	Coating of bioactive substances	Melt mixing / compression molding/ blown film extrusion	Reference
Rapid processing	Yes	Yes	[17]
Requires higher heat stability	No	Yes	[18], [19]
Uniformity of the films	Not always uniform	Not always uniform	[11], [15]
Effect on mechanical and physical properties of the film	Less likely	More likely	[11], [15]
Contact with food	Yes	No	[15]
Release of bioactive substances	More rapid	Slower or not happening	[20]
Antimicrobial activity	Higher	Lower	[11], [21] [22]

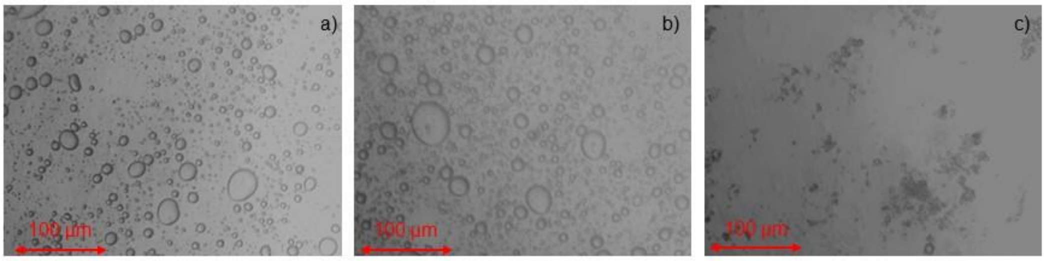

Figure S1. Optical microscope images of a) carvacrol-, b) thymol- and c) OLE-based mixtures (with MC/Tween 80).

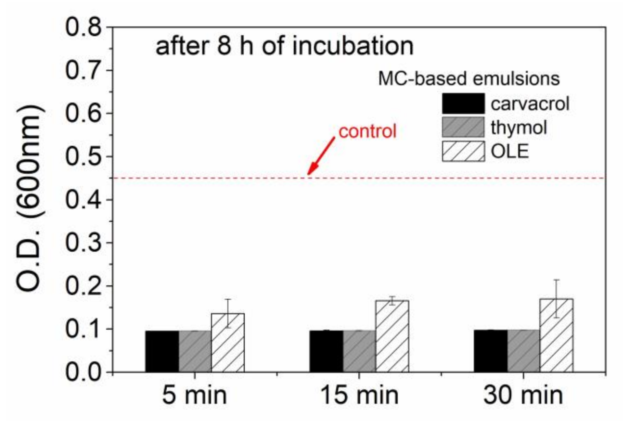

Figure S2: Antimicrobial properties of LDPE bioactive-coated films with MC-based emulsions after drying at 45 oC for 5 min, 15 min and 30min. Red line refers to the control, *E. coli* growth without any sample.

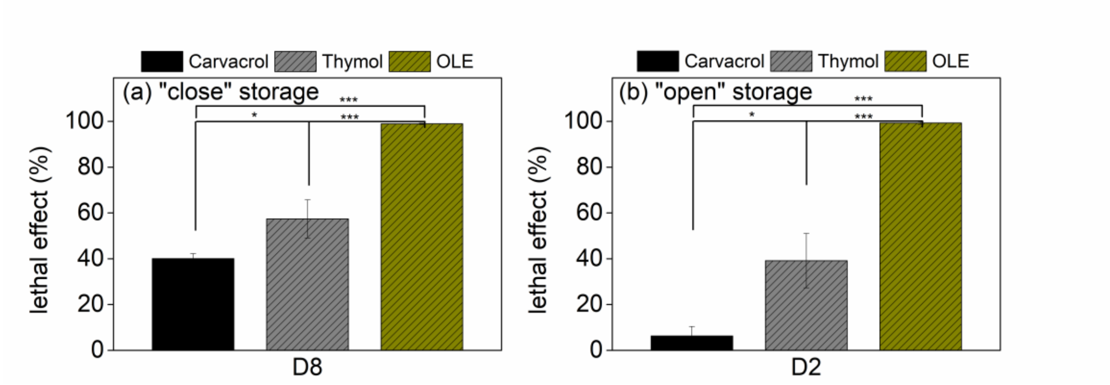

Figure S3: Comparison of the antimicrobial response of carvacrol-, thymol- and OLE-based films at critical time intervals for "close" and "open" storage conditions; a) "close" storage – day 8, b) "open" storage – day 2, against gram-negative bacteria *E. coli*. The lethal effect is defined as the percentage growth inhibition of *E. coli* cells compared to a control sample at the exponential growth phase (5 h) (* $p < 0.05$, ** $p < 0.01$, *** $p < 0.001$, **** $p < 0.0001$).

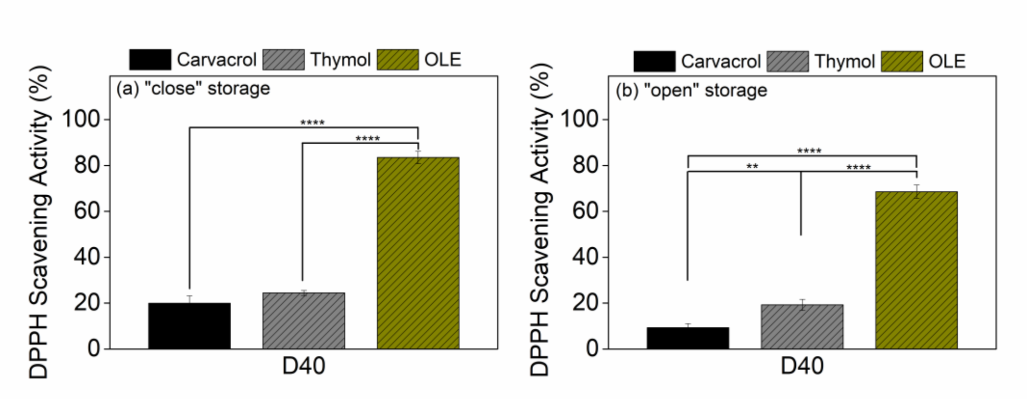

Figure S4: Comparison of the DPPH Scavenging activity of bioactive-coated films at critical time intervals for "close" and "open" storage conditions; a) "close" storage – day 40, b) "open" storage – day 40. (* $p < 0.05$, ** $p < 0.01$, *** $p < 0.001$, **** $p < 0.0001$).

Disclaimer/Publisher's Note: The statements, opinions and data contained in all publications are solely those of the individual author(s) and contributor(s) and not of MDPI and/or the editor(s). MDPI and/or the editor(s) disclaim responsibility for any injury to people or property resulting from any ideas, methods, instructions or products referred to in the content.
